# Infantile Hemangioma Presenting as Colocolic Intussusception in an Infant Case Report with Review of Pathologic Lead Points

**DOI:** 10.1155/2018/6494075

**Published:** 2018-06-24

**Authors:** Rehan Rais, Iván González, Jacqueline M. Saito, Louis P. Dehner

**Affiliations:** From the Lauren V. Ackerman Laboratory of Surgical Pathology, and Division of Pediatric Surgery, St. Louis Children's Hospital, Washington University Medical Center, St. Louis, MO, USA

## Abstract

Infantile hemangioma (IH) is one of the most common vascular anomalies of early childhood and is usually recognized in the first few weeks to months of life as a solitary cutaneous lesion. This report documents our experience with a GLUT-1 positive IH presenting as the pathologic lead point in a colocolic intussusception in a 10-week-old infant who had no skin lesions. Literature suggests approximately 2% of all children presenting with an intussusception require surgical intervention; however, an IH as the pathologic lead point is unique.

## 1. Introduction

The concurrence of intussusception and infantile hemangioma (IH) should not be entirely surprising, since the former is one of the most common causes of bowel obstruction, and the latter is the commonest cutaneous soft tissue tumor and vascular anomaly in infancy [1–4]. However, as our case illustrates, IH of the colon was the cause of a colocolic intussusception in a 10-week-old female infant which would appear to be an extraordinary coincidence in our review of the literature.

Intussusception is a well-known clinical entity to the pediatric surgeon as a cause of intestinal obstruction in children less than 5-years-old at presentation [1, 5]. In most children (90% or greater), the intussusception is an ileocolic invagination of the ileum into the cecum (85%–90% of cases) and is idiopathic in the absence of a pathologic lead point (in greater than 90% of cases) [1, 5, 6]. Most cases are managed without need for surgical resection [1, 2].

Infantile hemangioma as a cutaneous and/or subcutaneous lesion has a prevalence rate of 4.5% in infants at the age of 9 to 12 months [3, 4]. The incidence has increased over the past 30 years and has been associated with preterm birth and pregnancy complications [7, 8]. Visceral involvement, usually in the presence of five or more cutaneous IHs or diffuse infantile hemangiomatosis, is both rare and accompanied by multifocal or diffuse hemangiomatosis of the liver or in extraintestinal mucous membranes [9–11].

This report documents our experience with a localized IH of the colon. One earlier case in the literature is similar to ours, but the nature of the “capillary hemangioma” was not further characterized since immunohistochemistry for GLUT-1 was not performed [12, 13].

## 2. Case Report

### 2.1. Clinical Summary

A 10-week-old girl was the product of a full-term pregnancy which was complicated by maternal hypertension. No significant past medical or family history was present. The patient presented with a one day history of multiple episodes of nonbilious emesis, hematochezia, lethargy, and fussiness. On physical examination, the abdomen was diffusely tender to palpation with mild distension, and bowel sounds were audible. No cutaneous vascular lesions were noted at the time.

An abdominal ultrasound showed an apparent colocolic intussusception with no substantial interloop fluid collection, and color Doppler flow was demonstrated within the walls of the intussuscipiens and intussusceptum. A reduction of the intussusception was attempted with air contrast enema, and the intussusception was initially present at the rectosigmoid junction. With pressure maintained at less than 120 mm·Hg, the intussusception was reduced to the proximal descending colon, but the patient developed free intraperitoneal air apparent by fluoroscopy. During laparoscopic exploration, an intussusception was identified at the level of the distal descending colon, and fibrinous exudate was found along the descending colon, consistent with a perforation site. A segmental colonic resection with anastomosis was performed.

### 2.2. Pathologic Findings

**Figure 1:**
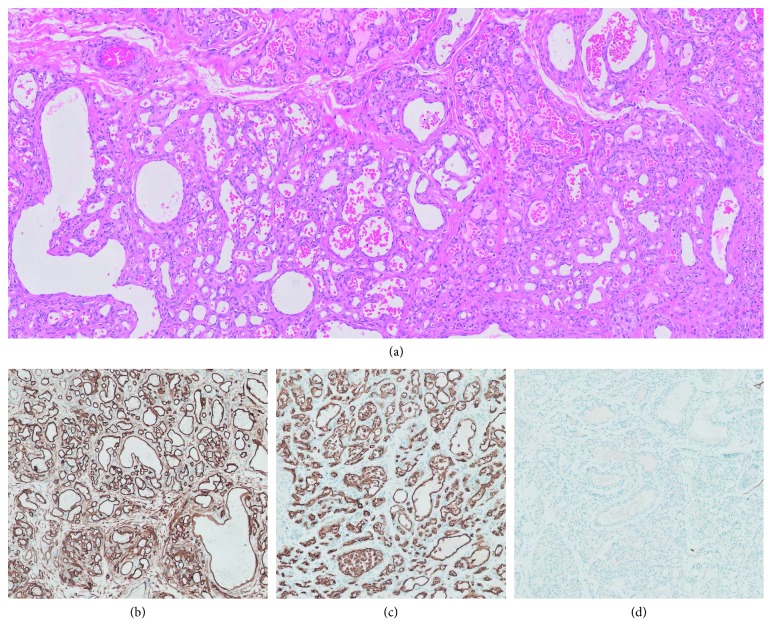
Histological sections showing lamina propria involved by a dense network of capillary-sized vascular spaces (a); immunohistochemistry was performed which shows endothelial positivity for, CD31 (b), GLUT-1 (c), and nonreactivity for D2-40 (d).

The resected segment of large intestine showed a telescoped segment of intestine. The intestinal wall measured from 0.2 to 0.5 cm. A minute gross perforation was identified; however, there was no evidence of a gross vascular malformation. Microscopic examination showed mucosal ischemic changes with vascular congestion. A vasoformative anomaly was present in the submucosa with involvement of the overlying mucosa where the lamina propria was occupied by a dense network of capillary-sized vascular spaces (Figure 1). Immunohistochemistry revealed that the endothelium was positive for vimentin, CD31 (Figure 1), CD34, GLUT-1 (Figure 1), and nonreactive for D2-40 (Figure 1).

## 3. Discussion

This report of a colocolic intussusception with a pathologic lead point (PLP) of an IH in the absence of any cutaneous vascular lesions documents a unique clinical event. The vast majority, 85–90%, of intussusceptions presenting in infancy are located in the ileocolic region and are regarded as idiopathic in the absence of PLP in 90% of cases which reflects our own experience in those children requiring intestinal resection. Colocolic intussusception as in our case represents only 2%-3% of all cases. The only other case comparable to our own presented in an almost 3-year-old boy who was intermittently symptomatic with abdominal pain, diarrhea, and eventual hematochezia and was found to have an ileocecal intussusception with a submucosal tumor measuring 2.5 cm in the cecum; immunohistochemical staining for GLUT-1, the sine qua non for establishing the diagnosis of IH, was not reported in this case, but the histologic features as illustrated and described could represent an IH. The mucosal and submucosal location of the IH in our case is similar in most respects to the cecal lesion in the prior case, but our patient was younger, and the IH was located in the descending colon.

Pathologic lead point in intussusception occurs far less commonly in children, based on individual case studies and case series, than in adults where it has been reported in as high as 90% of cases [6, 14–19]. A review of five pediatric series revealed that a PLP was found in less than 10% of cases (Table 1). The most common PLP in children is the Meckel's diverticulum accounting for 46% of cases. Polyps of one type or another were present in 13% of cases. In those studies specifying “multiple polyps,” there were examples of Peutz–Jeghers (PJ) polyps in the small intestine [6, 17, 18]; intussusception is reported in approximately 50%–70% of all cases of PJ syndrome [20]. Some have found that PLP-associated intussusceptions occur more commonly in “older children,” which is generally regarded as a child at or over 3 years of age. Literature suggests 90% of all intussusceptions in childhood present before the age of 3 years. The five studies cited in Table 1 are in general agreement that PLP-associated intussusceptions are diagnosed in children over 3 years of age and where Meckel's diverticulum is the most common PLP [6,15–18].

Vascular anomalies including vascular malformations and hemangiomas, as well as other types of benign tumors, are well documented as causes of intussusception in children. Blue rubber bleb syndrome is one of several syndromes with multifocal vascular malformations throughout the intestinal tract. The liver is the most common visceral site of IH, and it is often accompanied by multiple cutaneous IHs; the tumors in the liver are immunoreactive for GLUT-1. Multifocal hemangiomatosis with intestinal involvement is quite rare, but our case is unique with the colon as the only site of IH and as the PLP.

## Figures and Tables

**Table 1:** Pathologic lead points in intussusceptions. Review of five series.

	Banapour (15)	Rubinstein (16)	Ong (17)	Lin (6)	Wong (18)	*n* (%)
Meckel's diverticulum	7	2	27	32	8	76 (46)
Polyps, NOS	—	—	9	5	7	21 (13)
Duplication cyst	—	—	4	14	2	20 (12)
Lymphoma	—	—	5	4	1	10 (6)
Multiple polyps	—	3 (PJS)	3 (PJS, FAP)	3 (PJS)	—	9 (5)
Lymphoid hyperplasia	4	—	—	—	—	4 (2)
Vascular lesion	1	—	—	2 (hematoma)	—	3 (2)
Neuroendocrine tumor	—	—	2	—	—	2 (1)
Metastatic tumor	1 (EWS)	1 (WT)	—	—	—	2 (1)
Other	1	2 (HSP)	6	5	3	17 (10)
Total PLPs	14 (9%)	8 (6%)	56 (7%)	65 (7%)	21 (0.4%)	164 (2%)
Total cases	153	141	802	986	5,096	7,025

PJS = Peutz–Jeghers syndrome; FAP = familial adenomatous polyposis; EWS = Ewing sarcoma; WT = Wilms tumor; HSP = Henoch–Schönlein purpura; PLP = pathologic lead point.
